# Exosomal Adiponectin Plays a Role in Electroacupuncture Analgesia by Activating the Adiponectin Receptor 1 to Inhibit Pain‐Related Pathways in Mice Brain

**DOI:** 10.1155/prm/5402246

**Published:** 2026-09-12

**Authors:** Hsien-Yin Liao, Younbyoung Chae, Chien-Yi Ho, I.-Han Hsiao, Ming-Chia Lin, Yu-Ching Wang, Yi-Wen Lin

**Affiliations:** ^1^ College of Chinese Medicine, School of Post-Baccalaureate Chinese Medicine, China Medical University, Taichung 404328, Taiwan, cmu.edu.tw; ^2^ Acupuncture and Meridian Science Research Center, Kyung Hee University, Seoul 02453, South Korea, khu.ac.kr; ^3^ Department of Biomedical Imaging and Radiological Science, China Medical University, Taichung 404328, Taiwan, cmu.edu.tw; ^4^ Department of Family Medicine, China Medical University Hsinchu Hospital, Hsinchu 302233, Taiwan, cmu.edu.tw; ^5^ Physical Examination Center, China Medical University Hsinchu Hospital, Hsinchu 302233, Taiwan, cmu.edu.tw; ^6^ Department of Medical Research, China Medical University Hsinchu Hospital, Hsinchu 302233, Taiwan, cmu.edu.tw; ^7^ School of Medicine, College of Medicine, China Medical University, Taichung 404328, Taiwan, cmu.edu.tw; ^8^ Department of Neurosurgery, China Medical University Hospital, Taichung 404327, Taiwan, cmu.edu.tw; ^9^ Department of Nuclear Medicine, E-DA Hospital, I-Shou University, Kaohsiung 82445, Taiwan, isu.edu.tw; ^10^ Graduate Institute of Acupuncture Science, College of Chinese Medicine, China Medical University, Taichung 404328, Taiwan, cmu.edu.tw; ^11^ Chinese Medicine Research Center, China Medical University, Taichung 404328, Taiwan, cmu.edu.tw; ^12^ Office of Research and Development, Asia University, Taichung 413305, Taiwan, asia.edu.tw

**Keywords:** adiponectin, electroacupuncture, exosome, fibromyalgia, pPI3K, SIRT1

## Abstract

**Objective:**

Fibromyalgia (FM) is a difficult‐to‐cure disease, and finding effective pain management methods is crucial for providing clinicians and healthcare. Adiponectin (ADP) is considered a peripheral metabolic hormone, but its role in the central nervous system remains unknown, especially in pain management. Electroacupuncture (EA) has long been scientifically proven as an effective therapy for pain relief.

**Methods:**

We aimed to investigate the molecular mechanisms in the thalamus, somatosensory cortex, anterior cingulate cortex, and medial prefrontal cortex during intermittent cold stress (ICS)–induced FM in mice, and the inhibitory effects of 2‐Hz EA stimulation or intracerebral ventricle (ICV) injection of ADP on this FM model.

**Results:**

Results showed that the FM group significantly produced mechanical and thermal hyperalgesia (^∗^ *p* < 0.05, *n* = 9), while the FM + EA and FM + ADP groups showed significant reductions in mechanical and thermal hyperalgesia (^#^ *p* < 0.05, *n* = 9). The extracellular vesicles high mobility group box 1 and S100 calcium‐binding protein B (mediators in inflammation) were detected increased while ADP was found decreased, which can be reversed by EA but not ICV injection of ADP. Western blot analysis revealed a significant decrease in adiponectin receptor 1 (AdipoR1) and related signaling pathways after ICS‐induced FM hyperalgesia. However, encouragingly, in the FM + EA group, EA treatment effectively increased AdipoR1 expression. Similar results were observed in the FM + ADP group. This trend was also observed in its downstream pathways including APPL1, SIRT1, AMPKα, and PPARα. We further found a significant increase in pain‐related pPI3K–pAkt–pmTOR and pNFκB pathways in the FM group, and EA or ICV‐administered ADP treatment inhibited these increases.

**Conclusion:**

Our results confirm that 2‐Hz EA can treat FM pain, and its mechanism of action is through the activation of AdipoR1 and its downstream related molecular pathways.

## 1. Introduction

The incidence of fibromyalgia (FM) is 1%–8%, particularly affecting women aged 20 to 50, and the condition worsens with age. Currently, the U.S. Food and Drug Administration (FDA) has approved several medications for symptom relief, such as duloxetine, pregabalin, and mirtazapine, but their efficacy remains poor and they have significant side effects. Due to the lack of accurate symptom diagnosis and effective treatments, the prognosis for FM patients is expected to be even more severe. Treatment of FM is highly challenging due to difficulties in diagnosis, symptoms, and the lack of representative biomarkers; the most difficult aspect is determining the cause of the pain [1–3]. FM pain typically occurs extensively in the musculoskeletal system, ligaments, tendons, and soft tissues, especially in the shoulders, back, chest, arms, and legs. Neuroinflammation is also considered to be potentially associated with the progression of FM [4, 5]. Cordon et al. demonstrated that neuroinflammation is related to the progression of FM. They found that the number of neurons in the inner retinal layer of patients with FM was reduced due to neuroinflammation, and this was closely related to the severity of FM and quality of life [6]. In addition to medication, alternative therapies such as Tai Chi, exercise, or acupuncture can help patients relax muscles and reduce stress to alleviate pain [7, 8].

Adiponectin (ADP) is an active protein primarily secreted by white adipocyte cells. It can effectively enhance insulin sensitivity, burn white adipocyte cells, reduce inflammation, and protect blood vessels. ADP is abundant in the peripheral blood circulation. ADP receptors can be further divided into adiponectin receptor 1 (AdipoR1) and AdipoR2, which are seven‐transmembrane proteins that perform their functions by binding to the adipose tissue hormone ADP. AdipoR1 is a high‐affinity receptor that can bind to ADP and is mainly abundant in skeletal muscle and the brain. AdipoR2, on the other hand, is most abundant in the liver [9, 10]. A healthy diet and regular exercise to lose weight can significantly increase ADP concentration, which is beneficial to human health. ADP can also promote fatty acid oxidation in skeletal muscle and glucose absorption, thereby reducing glucose production in the liver. ADP has been shown to regulate fat and sugar metabolism throughout the body via blood flow and also has an inhibitory effect on vascular aging [11, 12]. Literature indicates that ADP can modulate the analgesic effect of electroacupuncture (EA) through the interaction between the peripheral blood circulation and the spinal cord. In a mouse model of inflammatory pain, ADP gene deletion inhibits the analgesic effect of acupuncture. Injecting ADP can mimic the analgesic effect of EA. These results show that the mechanism of EA analgesia is that EA releases ADP, which acts on ADP receptor 2 (AdipoR2). However, the activation of AdipoR2 can further activate the adaptor protein, phosphotyrosine interacting with PH domain and leucine zipper 1–5′‐adenosine monophosphate‐activated protein kinase–peroxisome proliferator–activated receptors (APPL1–AMPK–PPAR) signaling pathway [13]. ADP possesses anti‐neuroinflammatory, antioxidant, and metabolic regulating functions. ADP can suppress neuroinflammatory responses by inhibiting nuclear factor kappa‐light‐chain‐enhancer of activated B cells (NFκB), thereby reducing the release of proinflammatory cytokines such as tumor necrosis factor alpha (TNF‐α), interleukin‐6 (IL‐6), and IL‐1β [14, 15].

The pain signaling matrix is a set of brain regions including the thalamus, somatosensory cortex (SSC), anterior cingulate cortex (ACC), medial prefrontal cortex (mPFC), amygdala, insula, periaqueductal gray (PAG), and spinal cord that consistently respond to painful stimuli. Thalamus, SSC, ACC, mPFC, and hippocampus involved in ascending pathway. They received sensory and motor sensory information from peripheral nervous system. Hypothalamus and PAG more relate to the descending pathway that modulates the nociceptor. The ascending pain pathway transmits peripheral sensory information from the body through the spinal cord to the brain [16, 17]. These pathways typically use chains of three neurons to transmit sensory signals such as touch, pain, temperature, and body position to the cerebral cortex. The descending pain pathway is a set of neural circuits that run from top to bottom, enabling the brain to control and perceive the degree of pain. It transmits signals from the brain to the spinal cord [17, 18].

Acupuncture has been used for over 3000 years according to records. It is used to treat many diseases, especially in pain management. According to the World Health Organization, acupuncture has been clinically proven effective for hundreds of diseases. Acupuncture has significant clinical efficacy and minimal side effects and is currently widely used to treat symptoms such as back pain, lower back pain, toothache, headache, and FM [19–21]. Traditional acupuncture involves stimulating specific points with a sharp object or needle. Modern acupuncture uses a thin stainless steel needle inserted into specific points called acupoints. With technological advancements, electric current is often added to acupuncture, commonly known as EA. EA has been proven effective and reliable. A recent double‐blind randomized controlled trial demonstrated that acupuncture can significantly alleviate chronic pain and major depression comorbidity, with empirical scientific evidence showing that this is achieved by reducing inflammatory mediators [22]. EA has been shown to be quite effective for inflammatory pain, neuropathic pain, and FM [23–30]. Scientific research confirms that the mechanism of EA is through increasing the concentration of opioids, dopamine, and adenosine [31–35]. However, whether EA can increase ADP to activate ADP receptors in the neural circuits of the mouse brain to alleviate ICS‐induced FM remains to be observed.

This study primarily aimed to investigate the effects and mechanisms of FM and EA on neural circuits in the mouse brain. Our hypothesis was that ADP receptors in the thalamus, SSC, ACC, and mPFC of mice with FM were reduced. Our results showed that mice with intermittent cold stress (ICS)–induced FM exhibited mechanical and thermal hypersensitivity. 2‐Hz EA or intracerebroventricular (ICV) administration of ADP significantly suppressed these pain phenomena, consistent with our hypothesis. Western blot results revealed significantly reduced AdipoR1 protein levels in the abovementioned regions. Both 2‐Hz EA and ICV administration of ADP effectively reversed the low AdipoR1 protein levels. Similar phenomena were observed in the downstream pathways related to AdipoR1 including APPL1/SIRT1/AMPKα/PPARα. Further data showed a significant increase in the protein levels of the pain‐related molecule phosphorylated phosphoinositide 3‐kinase–phosphorylated protein kinase B– phosphorylated mechanistic target of rapamycin (pPI3K–pAkt–pmTOR) and phosphorylated nuclear factor kappa‐light‐chain‐enhancer of activated B cells (pNFκB), which was reversed by 2‐Hz EA and ICV administration of ADP. Our results indicate that EA can alleviate FM by increasing ADP receptors. We hope these excellent results will provide clinical guidance for the use of EA in the treatment of FM.

## 2. Materials and Methods

### 2.1. Animals and the Induction of FM Pain

All animal experiments used female C57BL/6 wild‐type mice (BioLASCO, Taipei, Taiwan), approximately 8–12 weeks old and weighing approximately 18–22 g. Mice were transported directly to the Laboratory Animal Center of China Medical University and housed in a specific pathogen‐free (SPF) environment. Housing conditions included a temperature of 25°C, a humidity of 60%, and a 12‐h light/12‐h dark cycle. All animal studies and care of live animals were approved and performed following the guidelines made by the Animal Ethics Committee of China Medical University, Taiwan (Approval No.: CMUIACUC‐2024‐076) and followed the Guidelines for the Use of Laboratory Animals (National Academy of Sciences Press). We made every effort to minimize mouse numbers and suffering. Mice of this sex were chosen because FM occurs in approximately 90% of cases in females, and gender differences in pain are increasingly confirmed in pain research; this issue should be systematically explored in the future. We used G∗Power 3.1.9.7 software to calculate the minimum sample size required for the experiments. To achieve a significance level of *α* = 0.05 and 80% statistical power, nine mice were required per group. The investigators were blinded to the group assignments and the review of the results. Mice were randomly assigned to four groups: a normal control group (normal group); ICS‐induced FM mice (FM group); EA‐treated FM mice (FM + EA group); and FM mice treated with intracerebral ventricle (ICV) ADP injection (FM + ADP group). We chose the ICS method to induce FM in mice. Mice were initially placed in a 4°C environment, while the normal control group mice were placed in a constant 25°C environment. The following morning at 10:00 a.m., the FM mice were placed in the 25°C normal environment for 30 min, followed by a return to the 4°C environment for 30 min. This entire process continued until 4:00 p.m. This process was repeated for 2 days.

### 2.2. EA

After anesthesia induction with 5% isoflurane, mice were placed in 1% gas via a head‐stuffed tube for maintenance. A pair of 1‐inch‐long acupuncture stainless steel needles (32G, Yu Kuang Chem. Ind. Corp., Taipei, Taiwan) were inserted bilaterally into the mouse ST36 acupoint, which is found 3–4 mm lower than the patella, between the fibula and tibia, and on the anterior side of the anterior tibial muscle. In addition, electrical stimulation was delivered through a constant square pulse at a depth of 3–4 mm with 1 mA intensity, 2‐Hz frequency, and 150 μs width for 20 min utilizing an electronic source from a Trio 300 stimulator (Ito, Tokyo, Japan). EA treatment sessions were conducted on Days 3 and 4 of the experiment.

### 2.3. Exosome Extraction

We used ExoQuick (System Biosciences, CA, USA) to isolate exosomes. First, serum was collected and centrifuged at 3000 × *g* for 15 min to remove cells and cell debris. The supernatant was transferred to a sterile container, and an appropriate amount of ExoQuick exosome precipitation solution was added. The mixture was thoroughly mixed by inverting or gently tapping the tube. The serum sample was refrigerated at 4°C for 30 min. During this time, the tube should not be rotated or mixed and should be kept upright. The ExoQuick/serum mixture was centrifuged at 1500 × *g* for 30 min at room temperature. After centrifugation, a white precipitate of exosomes appeared at the bottom of the container. The supernatant was aspirated. The remaining ExoQuick solution was centrifuged again at 1500 × *g* for 5 min. All liquid was aspirated, taking care not to touch the exosomes in the precipitate. The exosome precipitate was dissolved in 100–500 μL of a specific buffer solution. The precipitated exosomes were used immediately for experiments. We used CD9 as the exosome marker.

### 2.4. Nociceptive Behavioral Testing

Before measuring the pain behavior stimulus in mice, the mice were acclimatized to the environment for 3 days. Pain behavior was measured twice: once before ICS induction and again 4 days later. During the pain behavior test, the mice were placed in an acrylic box with a metal mesh bottom. The behavioral test was conducted in a dark, noise‐free environment at room temperature. First, the mice were tested using an electronic von Frey instrument (IITC Life Science Inc., California 91367, USA). During the test, the mice remained still and were not allowed to stand, sleep, scratch, or groom themselves. Each step was repeated three times, with 10–15 min between each step. Next, we used a Hargreaves’ instrument to measure the mice’s thermal pain response. We used an IITC Analgesiometer (IITC Life Science Inc., California 91367, USA) to measure the paw withdrawal latency to a radiant heat source. This device has an automatic setting to cut off the heat source after 20 s to avoid injury to the mouse’s hind paw.

### 2.5. Western Blot Analysis

The mice were sacrificed to extract their brains for protein extraction and stored them at −80°C. We used cold radioimmunoprecipitation (RIPA) lysis buffer (Fivephoton Biochemicals, San Diego, CA, USA) to extract all proteins. Its composition was 50 mM Tris–HCl pH 7.4, 250 mM NaCl, 1% NP‐40, 5 mM EDTA, 50 mM NaF, 1 mM Na3VO4, 0.02% NaN3, and a 1x protease inhibitor mixture (FC0070‐0001, Bionovas, Toronto, ON, Canada). The extracted proteins were separated by 8% SDS–Tris glycine gel electrophoresis and transferred to a polyvinylidene difluoride (PVDF) membrane. The PVDF membrane was incubated in 5% skim milk TBS‐T buffer (10 mM Tris pH 7.5, 100 mM NaCl, 0.1% Tween 20) and then incubated with primary antibody in 1% bovine serum albumin (BSA) TBS‐T buffer at room temperature for 1 h. The following first antibodies were used: AdipoR1 (∼45 kDa, 1:1000, Cat. No. 72674, Merck, Rahway, NJ, USA); APPL1 (∼80 kDa, 1:1000, Cat. No.: HPA011138, Merck, Rahway, NJ, USA); SIRT1 (∼120 kDa, 1:1000, Cat. No. S5196, Merck, Rahway, NJ, USA); AMPKα (∼63 kDa, 1:1000, Cat. No. 07‐350, Merck, Rahway, NJ, USA); PPARα (∼52 kDa, 1:1000, Cat. No. SAB2101852, Merck, Rahway, NJ, USA); pPI3K (∼125 kDa, 1:1000, Cat. No. PA5‐28070, Invitrogen, Waltham, MA, USA); pAkt (∼65 kDa, 1:1000, Cat. No. 9271, Cell Signaling, Danvers, MA, USA); pmTOR (∼289 kDa, 1:500, Cat. No. 05‐1592, Merck, Darmstadt, Germany); pNFκB (∼65 kDa, 1:1000, Cat. No. 481408, Merck, Rahway, NJ, USA). Peroxidase‐conjugated anti‐rabbit antibody or anti‐mouse antibody (1:5000) was used as a secondary antibody. Blot bands were visualized using a chemiluminescent substrate kit (PIERCE) and a LAS‐3000 Fujifilm (Fuji Photo Film Co., Ltd, Tokyo, Japan). Protein concentrations of the precise bands were determined using NIH Image J 1.54 h software (Bethesda, MD, USA). β‐Actin or α‐tubulin were used as internal controls. Percentages were calculated by dividing the treatment group value by the control group value.

### 2.6. Immunofluorescence Staining

Mice were first anesthetized with 5% isoflurane and injected cardiacally with 0.9% saline, followed by an injection of 4% paraformaldehyde. Brain tissue was immediately removed and fixed in 4% paraformaldehyde for 3 days at 4°C. The brain tissue was then further placed in 30% sucrose solution and stored overnight at 4°C. We used a tissue slicer to cut the brain tissue into 16‐μm‐thick slices. The brain slice sections were blocked for 1 h at room temperature with 0.1% Triton X‐100/PBS containing 0.02% sodium azide and 3% BSA. The samples were then incubated overnight with primary antibodies (anti‐AdipoR1 and anti‐SIRT1 antibodies). Sections were washed three times with 0.05% Triton X‐100/PBS and then incubated with secondary antibodies in 0.1% Triton X‐100/PBS containing 3% BSA for 2 h at room temperature. Secondary antibodies used were as follows: (1:500), 488‐conjugated AffiniPure donkey anti‐rabbit IgG (H + L) and 594‐conjugated AffiniPure donkey anti‐goat IgG (H + L) for 2 h at room temperature before fixation with cover slips for immunofluorescence visualization.

### 2.7. Intracerebroventricular Injection

We first anesthetized the mice with isoflurane, fixed their heads to a stereotaxic apparatus, and inserted a cannula into the ventricle. The stereotaxic cannula (23 gauge 2 mm stainless steel, Doric, QC, Canada) was positioned 0.5 mm from the anteroposterior axis, approximately 1 mm from the medial‐lateral axis, and approximately 2.5 mm from the dorsoventral axis, subcortical. It was then fixed to the skull with dental cement. We inserted the cannula into the ventricle and connected it to a Hamilton syringe through a PE tube (PE10, Portex, Kent, UK). Using an injection pump (KD Scientific, Shanghai, China), 0.1 μg of ADP (1 μL/ventricle) was injected into the ventricle over 5 min. After inoculation, the tube was left in place for 2 min to allow ADP diffusion.

### 2.8. Statistical Analysis

We used SPSS 21.0 statistical software to perform between‐group differences. All statistical data are presented as mean ± standard error of the mean (SEM). Shapiro–Wilk test was performed to examine the normality of the results. Differences between groups were tested by one‐way analysis of variance (ANOVA) and Tukey’s post hoc test was performed. *p* < 0.05 was considered statistically significant.

## 3. Results

### 3.1. 2‐Hz EA or Intraventricular Administration of ADP Significantly Treats ICS‐Induced Chronic Mechanical and Thermal Hyperalgesia in Mice

**FIGURE 1:**
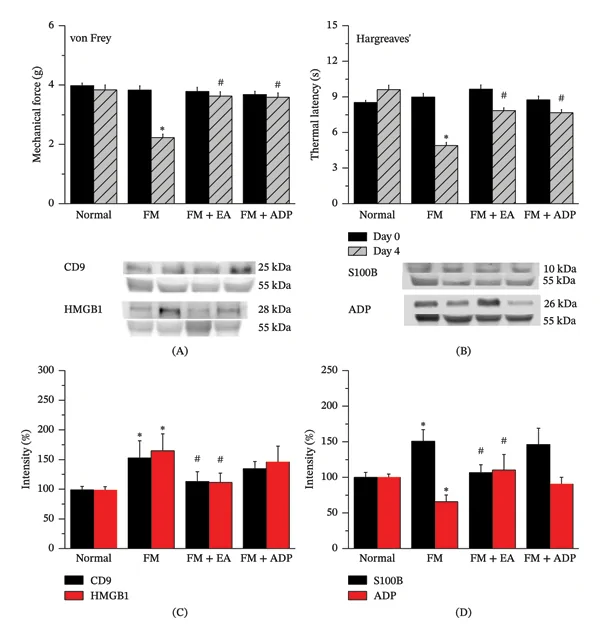
We measured the effects of ICS‐induced FM on mechanical and thermal hyperalgesia in mice on Days 0 and 4 using von Frey and Hargreaves’ tests. (A) Mechanical hyperalgesia threshold in mice; (B) thermal hyperalgesia withdrawal latency in mice; (C) Western blots of plasma EV lysates confirmed that the vesicles contained CD9 and HMGB1; (D) Western blots of plasma EV lysates showed that the vesicles contained S100B and ADP. Responses of mice in the normal group, ICS‐induced FM group, ICS‐induced FM mice treated with EA, and FM mice treated with ICV‐ADP injection. ^∗^ *p* < 0.05 indicates statistically significant difference compared to the normal group. ^#^ *p* < 0.05 indicates statistically significant difference compared to the FM group. *n* = 9 per group.

To demonstrate the detailed mechanism of EA in treating FM, we first induced FM in mice using ICS. We compared the mechanical pain responses in four different groups of mice on the von Frey test. Before ICS induction, the mechanical responses of the mice were similar and showed no statistical difference. Normal mice, without ICS induction, exhibited normal mechanical pain responses on Day 4 (Figure 1A, gray column, Day 4: 3.84 ± 0.16 g, *n* = 9). After ICS‐induced FM, the mice showed significant mechanical hyperalgesia on Day 4 (Figure 1A, gray column, Day 4: 2.23 ± 0.11 g, ^∗^ *p* < 0.05, *n* = 9). 2‐Hz EA after ICS induction effectively inhibited mechanical hyperalgesia (Figure 1A, gray column, Day 4: 3.63 ± 0.15 g, ^#^ *p* < 0.05, *n* = 9). Reduced mechanical hyperalgesia was observed in the ICV administration of ADP group mice on Day 4, indicating that ADP can treat mechanical hyperalgesia (Figure 1A, gray column, Day 4: 3.59 ± 0.14 g, ^#^ *p* < 0.05, *n* = 9). We then used the Hargreaves’ test to measure thermal hyperalgesia in mice; the trend of thermal pain was similar to that of mechanical hyperalgesia. All groups of mice showed similar thermal hyperalgesia responses at baseline, with no statistically significant differences. The latency of thermal hyperalgesia was significantly shorter in the FM group mice (Figure 1B, gray column, Day 4: 4.89 ± 0.26 s, ^∗^ *p* < 0.05, *n* = 9). Treatment with 2‐Hz EA significantly reduced thermal hyperalgesia in FM mice (Figure 1B, gray column, Day 4: 7.85 ± 0.24 s, ^#^ *p* < 0.05, *n* = 9). ICV administration of ADP to mice resulted in lower levels of thermal pain (Figure 1B, gray column, Day 4: 7.67 ± 0.26 s, ^#^ *p* < 0.05, *n* = 9).

We then confirmed using Western blotting that the extracellular vesicles (EVs) marker protein CD9 was significantly increased in plasma EV lysates in the FM group (Figure 1C, 152.47% ± 29.28%, black column, ^∗^ *p* < 0.05, *n* = 4), and a similar trend was observed in HMGB1 protein lysate (Figure 1C, 164.71% ± 28.43%, red column, ^∗^ *p* < 0.05, *n* = 4). These increasing trends decreased after mice received 2‐Hz EA (Figure 1C, 112.84% ± 16.61% and 111.40% ± 15.83%, black and red columns, ^#^ *p* < 0.05, *n* = 4). However, ICV administration of ADP did not alleviate the increase in CD9 and HMGB1 in plasma (Figure 1C, 134.67% ± 11.83% and 146.02% ± 26.62%, black and red columns, *p* > 0.05, *n* = 4). Next, we observed the expression levels of S100B‐containing proteins in EVs, and the results showed that the level of S100B increased in the FM group (Figure 1D, 150.73% ± 16.38%, black column, ^∗^ *p* < 0.05, *n* = 4). However, the expression level of ADP‐containing proteins in EVs decreased in FM mice (Figure 1D, 65.82% ± 9.36%, red column, ^∗^ *p* < 0.05, *n* = 4). These trends were reversed by 2‐Hz EA, while the ICV administration of ADP had no effect (Figure 1D).

### 3.2. 2‐Hz EA or Intraventricular Administration of ADP to Mice Enhanced the Protein Expression of AdipoR1 in the Thalamus Region, Thereby Inhibiting the Pain Pathway

**FIGURE 2:**
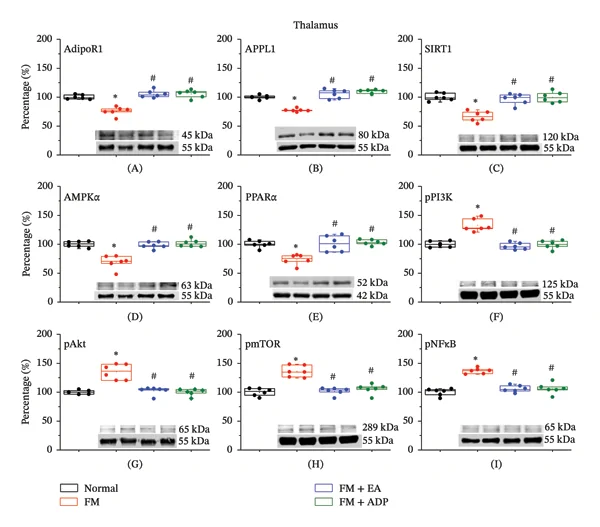
Western blot was used to analyze the expression levels of AdipoR1‐related signaling pathways and pain‐related molecules in the mouse thalamus. We examined the expression levels in the normal group, FM group, FM + EA group, and FM + ADP group. (A) AdipoR1, (B) APPL1, (C) SIRT1, (D) AMPKα, (E) PPARα, (F) pPI3K, (G) pAkt, (H) pmTOR, (I) pNFκB. ^∗^ *p* < 0.05 vs. normal group. ^#^ *p* < 0.05 vs. FM group. *n* = 6 per group.

We first used Western blotting to analyze the expression of ADP receptors and pain pathway‐related proteins in the thalamus region of FM mice. We found that the expression of AdipoR1 receptor protein in the thalamus region of FM mice was significantly low (Figure 2A, ^∗^ *p* < 0.05, *n* = 6); however, 2‐Hz EA treatment increased AdipoR1 protein expression (Figure 2A, ^#^ *p* < 0.05, *n* = 6). We further administered ADP via ICV to the mice, which increased AdipoR1 protein levels (Figure 2A, ^#^ *p* < 0.05, *n* = 6). We further analyzed the protein expression of the AdipoR1‐binding protein APPL1. The protein level of APPL1 in the thalamus region of FM mice was low (Figure 2B, ^∗^ *p* < 0.05, *n* = 6); however, 2‐Hz EA treatment or ICV injection of ADP effectively increased AdipoR1 protein expression (Figure 2B, ^#^ *p* < 0.05, *n* = 6). We hope to further investigate whether SIRT1 is involved in the development of FM. The significantly lower levels of SIRT1 protein in the thalamus region of FM mice indicate its involvement in the induction of FM (Figure 2C, ^∗^ *p* < 0.05, *n* = 6); however, this phenomenon was alleviated by 2‐Hz EA or ICV injection of ADP (Figure 2C, ^#^ *p* < 0.05, *n* = 6). Exploring downstream pathways, AMPKα and PPARα in the thalamus region of FM mice were significantly reduced due to ICS‐induced FM (Figure 2E, ^∗^ *p* < 0.05, *n* = 6). 2‐Hz EA or ICV injection of ADP significantly increased the protein levels of these substances in FM mice (Figure 2D,E, ^#^ *p* < 0.05, *n* = 6). To investigate whether pain‐related hormones are involved in this FM model and the role of ADP, our results showed that the pPI3K–pAkt–pmTOR pain pathway was significantly increased in the thalamus of FM mice (Figure 2F–H, ^∗^ *p* < 0.05, *n* = 6), and 2‐Hz EA or ICV injection of ADP stably reversed this decreasing trend (Figure 2F–H, ^#^ *p* < 0.05, *n* = 6). We further investigated whether the transcription factor pNFκB was affected by this model; we found that pNFκB in the thalamus region of FM mice was significantly increased (Figure 2I, ^∗^ *p* < 0.05, *n* = 6), but this phenomenon was mitigated by 2‐Hz EA or ICV injection of ADP (Figure 2I, ^#^ *p* < 0.05, *n* = 6).

### 3.3. 2‐Hz EA or Intraventricular Administration of ADP Enhances AdipoR1 Receptors, Thereby Alleviating Pain Signaling Pathways in the SSC

**FIGURE 3:**
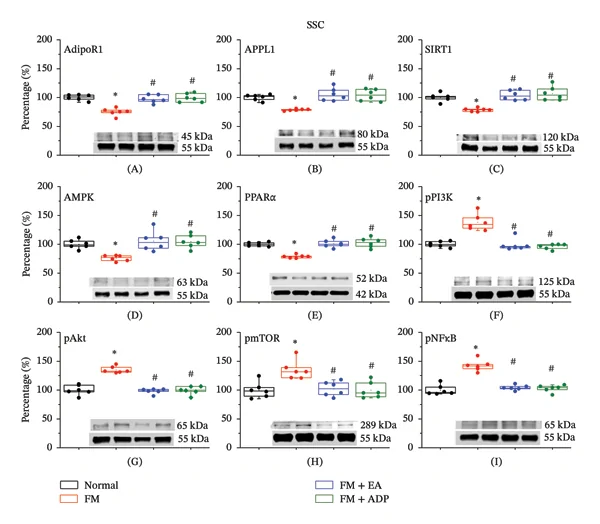
Western blotting was performed to analyze the protein levels of the AdipoR1 pathway and pain molecules in mouse SSC. We measured expression levels in the normal group, FM group, FM + EA group, and FM + ADP group. (A) AdipoR1, (B) APPL1, (C) SIRT1, (D) AMPKα, (E) PPARα, (F) pPI3K, (G) pAkt, (H) pmTOR, (I) pNFκB. ^∗^ *p* < 0.05 vs. normal group. ^#^ *p* < 0.05 vs. FM group. *n* = 6 per group.

To investigate the effects of ICS‐induced FM on AdipoR1 protein and pain signaling pathways in the SSC region, we performed the protein analysis of the AdipoR1 signaling pathway. We found that AdipoR1 expression was relatively low in the SSC region of FM mice (Figure 3A, ^∗^ *p* < 0.05, *n* = 6), indicating reduced analgesic capacity. 2‐Hz EA significantly increased AdipoR1 protein levels in FM mice (Figure 3A, ^#^ *p* < 0.05, *n* = 6). However, ICV administration of ADP had a similar effect (Figure 3A, ^#^ *p* < 0.05, *n* = 6). The protein level of APPL1, the AdipoR1‐binding protein, was significantly reduced in the SSC region of FM mice (Figure 3B, ^∗^ *p* < 0.05, *n* = 6), and this phenomenon was successfully reversed by 2‐Hz EA or ICV administration of ADP (Figure 3B, ^#^ *p* < 0.05, *n* = 6). We hypothesized that the downstream molecule SIRT1 of AdipoR1 is involved in this FM model, and the results indicated that the reduced SIRT1 protein level in the SSC cells of FM mice led to pain (Figure 3C, ^∗^ *p* < 0.05, *n* = 6). However, 2‐Hz EA or ICV administration of ADP significantly reversed this result in FM mice (Figure 3C, ^#^ *p* < 0.05, *n* = 6). Further analysis of downstream pathway molecules revealed relatively low levels of AMPKα and PPARα protein expression in FM mouse SSC cells (Figure 3D,E, ^∗^ *p* < 0.05, *n* = 6). Treatment with 2‐Hz EA or intraventricular ADP in FM mice increased AMPKα and PPARα expression (Figure 3D,E, ^#^ *p* < 0.05, *n* = 6). We then focused on the pain‐related signaling pathway pPI3K–pAkt–pmTOR. The results showed a significant increase in pPI3K–pAkt–pmTOR protein in the SSC nuclei of FM mice (Figure 3F–H, ^∗^ *p* < 0.05, *n* = 6), but 2‐Hz EA or ICV‐ADP treatment reduced this overexpression (Figure 3F–H, ^#^ *p* < 0.05, *n* = 6). Finally, we observed pNFκB expression in the SSC regions of FM mice. We found a significant increase in pNFκB in the SSC nuclei of FM mice (Figure 3I, ^∗^ *p* < 0.05, *n* = 6). 2‐Hz EA or ICV‐ADP treatment not only treated FM but also significantly reduced the significant increase in pNFκB (Figure 3I, ^#^ *p* < 0.05, *n* = 6).

### 3.4. The AdipoR1 Signaling Pathway Is Reduced in the Anterior Cingulate Cortex and mPFC of Mice, Which Is Associated With FM Formation

**FIGURE 4:**
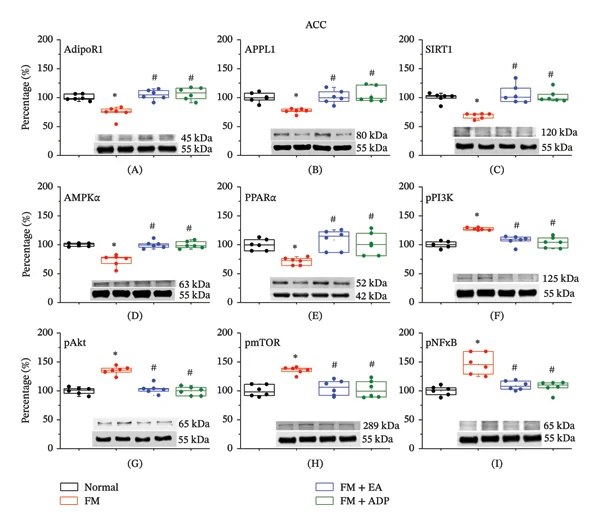
We measured the protein levels of the AdipoR1 downstream molecular pathway and pain‐related hormones in the ACC of mice. We measured the following groups: (A) AdipoR1, (B) APPL1, (C) SIRT1, (D) AMPKα, (E) PPARα, (F) pPI3K, (G) pAkt, (H) pmTOR, (I) pNFκB. ^∗^ *p* < 0.05 vs. normal group. ^#^ *p* < 0.05 vs. FM group. *n* = 6 per group.

**FIGURE 5:**
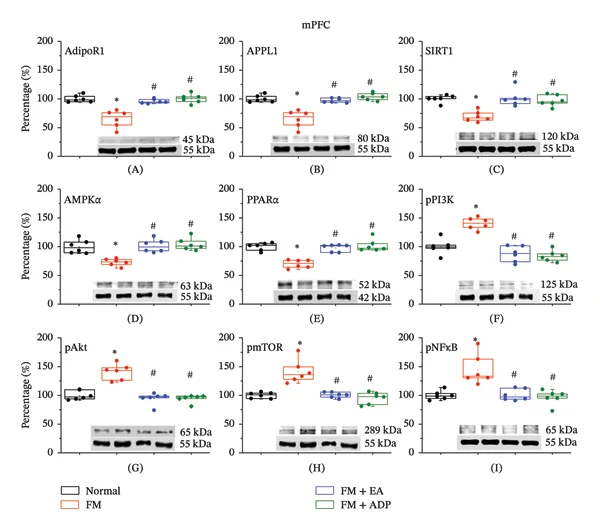
We then examined the protein levels of AdipoR1 downstream molecular mechanisms and pain‐related factors in mouse mPFC. We measured the following groups: (A) AdipoR1, (B) APPL1, (C) SIRT1, (D) AMPKα, (E) PPARα, (F) pPI3K, (G) pAkt, (H) pmTOR, (I) pNFκB. ^∗^ *p* < 0.05 vs. normal group. ^#^ *p* < 0.05 vs. FM group. *n* = 6 per group.

To determine whether EA or ICV administration of ADP could improve AdipoR1 receptor expression in the ACC and mPFC regions of mice, we performed Western blot analysis. AdipoR1 is expressed in the normal ACC and mPFC regions, and its expression is reduced after ICS‐induced FM (Figures 4–5A, ^∗^ *p* < 0.05, *n* = 6). 2‐Hz EA significantly increased AdipoR1 levels, and similar AdipoR1 levels were observed in the ACC and mPFC regions of the FM + ADP group as in the normal group (Figures 4–5A, ^#^ *p* < 0.05, *n* = 6). We also evaluated the effects of EA on APPL1 and SIRT1. Western blot analysis showed reduced APPL1 and SIRT1 expression in the FM group (Figure 4B,C, Figure 5B,C, ^∗^ *p* < 0.05, *n* = 6). FM mice showed significantly increased APPL1 and SIRT1 expression after 2‐Hz EA or ICV administration of ADP (Figure 4B,C, Figure 5B,C, ^#^ *p* < 0.05, *n* = 6). Further investigation down the AdipoR1 pathway revealed that AMPKα and PPARα were also reduced in the ACC and mPFC regions of FM mice (Figure 4D,E, Figure 5D,E, ^∗^ *p* < 0.05, *n* = 6). However, the analgesic effect of 2‐Hz EA or ICV on ADP is achieved by increasing the levels of these proteins (Figure 4D,E, Figure 5D,E, ^#^ *p* < 0.05, *n* = 6). In FM mice, the expression of the pain‐related kinase pPI3K–pAkt–pmTOR increases in the ACC and mPFC regions in response to pain Figure 4F–H, Figure 5F–H, ^∗^ *p* < 0.05, *n* = 6). The analgesic effect of 2‐Hz EA or ICV on ADP treatment is achieved by reducing the pPI3K–pAkt–pmTOR signaling pathway (Figure 4F–H, Figure 5F–H, ^#^ *p* < 0.05, *n* = 6). Similar results were also observed in the transcription factor pNFκB. These data suggest that AdipoR1 plays a crucial role in this FM mouse model and indicate that EA may modulate the AdipoR1 pathway.

### 3.5. Effects of FM on AdipoR1 and SIRT1 Protein Expression and the Regulatory Effect of EA or ICV Injection of ADP: Immunofluorescence Results

**FIGURE 6:**
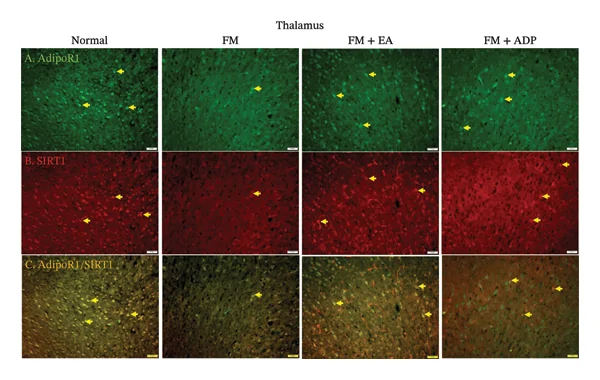
Immunofluorescence intensities of AdipoR1, SIRT1, and colabeled markers in the mouse thalamus. (A) AdipoR1, (B) SIRT1, and (C) colabeled staining (red, green, or yellow) in the mouse thalamus. AdipoR1, SIRT1, and colabeled marker levels were significantly attenuated in FM mice, while these markers were significantly increased in EA‐treated or ICV injection of ADP. All groups *n* = 3.

**FIGURE 7:**
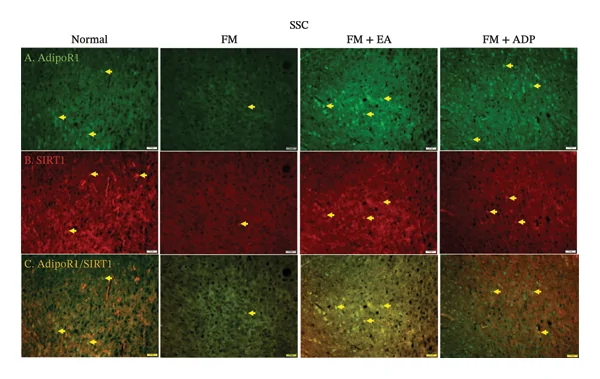
Immunofluorescence intensities of AdipoR1, SIRT1, and colabeled markers in the mouse SSC. (A) AdipoR1, (B) SIRT1, and (C) colabeled staining (red, green, or yellow) in the mouse SSC. AdipoR1, SIRT1, and colabeled marker levels were significantly attenuated in FM mice, while these markers were significantly increased in EA‐treated or ICV injection of ADP. All groups *n* = 3.

We then used immunofluorescence staining to analyze the effects of FM on the expression of AdipoR1 and SIRT1 proteins. Compared with normal mice, the fluorescence intensity of AdipoR1 in the thalamus of FM mice was significantly reduced, indicating that pain causes a decrease in its protein levels. EA treatment and ICV injection of ADP both increased its expression, suggesting an analgesic effect (Figure 6A, *n* = 3). The detection of SIRT1 protein levels showed that the fluorescence intensity of SIRT1 in the thalamus of FM mice was significantly lower. EA treatment or ICV injection of ADP both effectively increased the fluorescence intensity of SIRT1, suggesting an analgesic effect (Figure 6B, *n* = 3). Colabeling of the expression of these two proteins revealed a common expression region, confirming that they play a common pathway (Figure 6C, *n* = 3). The same trend was also found in the SSC brain region of mice (Figures 7).

## 4. Discussion

This study provides evidence that ICS‐induced FM occurs in mice. 2‐Hz EA or ICV administration of ADP effectively reduced mechanical and thermal hyperalgesia in mice. The EVs HMGB1 and S100B were detected increased, while ADP was found decreased, which can be reversed by EA but not ICV injection of ADP. The ascending pain pathway in the thalamus is a crucial integrative hub in the brain network. First, we found a strong reduction in AdipoR1 receptors in the thalamus, a similar finding in the SSC, ASS, and mPFC regions, indicating that the thalamus is connected to multiple pain cortical regions. The same trend was observed in downstream pathways such as APPL1, SIRT1, AMPKα, and PPARα. Conversely, pain‐related pathways such as pPI3K–pAkt–pmTOR showed significant increases in these regions, and the same trend was observed in pNFκB protein expression. 2‐Hz EA or ICV administration of ADP effectively improved these findings.

The incidence of chronic postoperative pain syndrome (CPSP) after fracture surgery is as high as 50%, and evidence suggests that it may be related to inflammatory responses. A recent prospective clinical study analyzed serum samples from 30 patients with traumatic fractures requiring surgery. The results showed a significant increase in RANTES/CCL5 (chemokine ligand 5) levels in CPSP patients as early as postoperative Day 1, indicating an enhanced inflammatory response. However, patients who developed CPSP 1 year postoperatively had relatively higher levels of resistin and ADP. These reports indicate that ADP and resistin are considered novel biomarkers for the development of CPSP [36]. One study indicated that the concentration of cartilage oligomeric matrix protein was significantly higher in combat‐injured patients compared to the uninjured group. Combat‐injured patients with knee pain also had relatively lower levels of ADP, which is related to pain [37].

ADP has a wide range of functions in brain tissue, activated through binding to its specific receptor, AdipoR1. These functions include anti‐inflammatory, antiapoptotic, and analgesic effects. Studies by Iannitti et al. found that in rats with induced peripheral inflammation, ADP, when injected into the spinal cord, produces analgesic and anti‐inflammatory effects via the AdipoR1 receptor. Their results further confirmed that preadministration of ADP effectively inhibits thermal hyperalgesia induced by Carrageenan injection [38]. Rats with obese have inflammatory hyperalgesia accompanied by decreased levels of ADP in the spinal cord level. Low concentrations of ADP in the blood have been observed in patients with chronic headaches, osteoarthritis pain, and postoperative pain [39]. Ning’s team discovered that EA can inhibit CFA‐induced inflammatory pain. They further confirmed that the effect of EA in treating inflammatory pain is weakened after ADP gene knockout and that intrathecal injection of ADP has the analgesic effect of EA. Using siRNA technology to inhibit AdipoR in the spinal cord, they demonstrated that EA mainly acts on the AdipoR2–AMPK pathway through ADP release, as EA increases the concentration of ADP in the spinal cord through blood circulation [38]. Our results showed a significant increase in EVs containing CD9, HMGB1, and S100B in the plasma of the FM group. However, the expression level of ADP‐containing protein in EVs was decreased in the FM group. EA attenuated the enhancing effects of CD9, HMGB1, and S100B. Furthermore, EA increased the content of ADP‐containing protein in EVs. ICV injection of ADP had no effect on these results. These phenomena may indicate that EA can affect both the peripheral and central nervous systems, but ICV administration of ADP can only alter the central nervous system.

ADP has been reported to have a protective effect in knee osteoarthritis. Scientific reviews have indicated that increased blood ADP levels are associated with osteophytes, narrowing of the joint space, and higher radiographic scrofula. ADP has also been reported to reduce TNFα levels by converting macrophages from M1 to M2 type. In addition, increased ADP can reliably activate the tissue inhibitor of MMP‐2, which can further attenuate the IL‐1β‐activated overexpression of MMP‐13 [40]. Ma et al.’s research used a virtual screening repositioning campaign to screen a large number of compounds and derivatives with different structures. They discovered that (+)‐MML1017 has a high binding affinity for AdipoR1 and AdipoR2. Further Western blot results showed that (+)‐MML1017 can phosphorylate AMPK by activating AdipoR1 in neurons. They also confirmed that (+)‐MML1017 can prevent the functional impairment of motor neurons under hyperglycemic conditions [41]. Malheiros et al. pointed out that inducing obesity in rats increased mechanical and thermal hyperalgesia, accompanied by increased concentrations of systemic inflammatory cytokines such as TNF‐α, IL‐1β, IL‐6, and leptin. Conversely, anti‐inflammatory cytokines such as ADP and IL‐10 decreased. Their further analysis revealed that obesity comorbid with osteoarthritis produced the highest levels of inflammatory cytokines and pain scores, while anti‐inflammatory factors decreased to the lowest levels. These results indicate that obesity‐induced ADP alters the pain threshold caused by inflammation [42]. Blockade of the AdipoR1 signaling pathway attenuated inflammation and improved joint damage in collagen‐induced arthritis [43]. A recent study has indicated that the rs12045862 SNP variant in the AdipoR1 gene is associated with postoperative pain, but not with cancer pain. This may be due to functional changes in the AdipoR1 signaling pathway affecting the inflammatory process [44]. EA also effectively ameliorates traumatic brain injury by reducing neuroinflammation and activating the AdipoR1 pathway [45].

## 5. Conclusion

**FIGURE 8:**
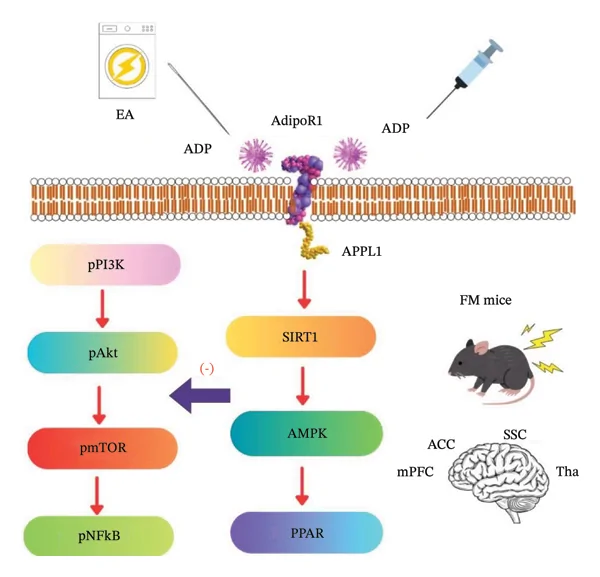
We utilize this illustration to summarize the findings of this study. EA or ICV‐ADP can inhibit FM pain in mice by inducing a series of molecular mechanisms via AdipoR1. These molecular mechanisms can be observed in thalamus, SSC, ACC, and mPFC.

In this regard, our results confirm that ICS can stably induce FM pain in mice. These mechanical and thermal hyperalgesia can be alleviated by 2‐Hz EA or ADP administration via ICV. Our investigation into the role and mechanism of AdipoR1 in the ascending pathway in this FM mouse model showed that AdipoR1 was reduced in brain regions such as thalamus, SSC, ACC, and mPFC in the ascending pathway during the FM pain pattern. Similar findings were observed in the expression levels of related AdipoR1 signaling pathway molecules such as APPL1, SIRT1, AMPK, and PPAR proteins. However, 2‐Hz EA or ICV administration of ADP significantly improved these reductions. We further note that pPI3K–pAkt–pmTOR and pNFκB were significantly increased in these regions during this pain pattern. Similarly, EA or ICV administration of ADP effectively alleviated these overexpressions. This evidence clearly confirms that EA is effective in treating FM pain in mice primarily through the AdipoR1 signaling pathway (Figure 8). We expect these results to provide guidance for the clinical treatment of FM.

## 6. Limitations

The limitation of this ICS model is that it cannot reflect factors such as chronic fatigue, rheumatic immunity, and family history in FM. Although ICS can induce FM‐like pain, it cannot assess broader FM features such as autonomic dysfunction, fatigue, stiffness, and sleep disorders. Another limitation is that we only analyzed AdipoR1 receptors and cannot rule out the involvement of other receptors. Clinical trials are needed to validate our findings. Focusing only on the four regions mentioned above is insufficient. We must investigate other pain‐processing structures (such as the insula, amygdala, PAG matter, or spinal cord) in the near future.

## Author Contributions

Hsien‐Yin Liao, Younbyoung Chae, Chien‐Yi Ho, I.‐Han Hsiao, and Ming‐Chia Lin conceptualization, methodology, software, data curation, writing–original draft, visualization, and investigation. Yu‐Ching Wang and Yi‐Wen Lin: supervision, validation, and writing–review and editing.

## Funding

This work was supported by the National Science and Technology Council: NSTC 114‐2314‐B‐039‐031, NSTC 114‐2314‐B‐039‐087. China Medical University Hsinchu Hospital: CMUHCH‐CMU‐115‐008, and the “Chinese Medicine Research Center, China Medical University” from The Featured Areas Research Center Program within the framework of the Higher Education Sprout Project by the Ministry of Education (MOE) in Taiwan.

## Ethics Statement

The animal study protocol was approved by the Institute of Animal Care and Use Committee of China Medical University (Approval No.: CMUIACUC‐2024‐076, 12/22/2023).

## Conflicts of Interest

The authors declare no conflicts of interest.

## Data Availability

The data that support the findings of this study are available on request from the corresponding author. The data are not publicly available due to privacy or ethical restrictions.
